# Using Digital Communication Technology to Increase HIV Testing Among Men Who Have Sex With Men and Transgender Women: Systematic Review and Meta-Analysis

**DOI:** 10.2196/14230

**Published:** 2020-07-28

**Authors:** Vanessa Veronese, Kathleen Elizabeth Ryan, Chad Hughes, Megan SC Lim, Alisa Pedrana, Mark Stoové

**Affiliations:** 1 Disease Elimination Program Burnet Institute Melbourne Australia

**Keywords:** digital technology, men who have sex with men, transgender women, HIV testing, HIV prevention

## Abstract

**Background:**

HIV continues to disproportionately affect men who have sex with men (MSM) and transgender women (TW). Undiagnosed HIV is a major driver of HIV transmission rates, and increasing the uptake of regular HIV testing and facilitating timely initiation of HIV treatment is a global HIV prevention priority. However, MSM and TW experience a range of barriers that limit their access to testing and other prevention services. Given their growing ubiquity, digital communication technologies are increasingly being used to support HIV prevention efforts, and a growing number of studies have trialed the use of digital technology to promote HIV testing among MSM and TW.

**Objective:**

We undertook a systematic review and meta-analysis to assess the impact of digital communication technology on HIV testing uptake among MSM and TW. Subanalyses aimed to identify the features and characteristics of digital interventions associated with greater impact.

**Methods:**

A systematic literature review was undertaken using select databases and conference repositories. Studies describing the use of a digital technology—internet-enabled devices, including phones, tablets, and computers—to increase HIV testing uptake among MSM or TW using either randomized or observational cohort design with measurement of HIV testing rates measured pre- and postintervention, and published in English between 2010 and 2018 were included. Pooled effect estimates were calculated using a random effects meta-analysis. Subanalyses calculated effect estimates grouped by selected features of digital interventions.

**Results:**

A total of 13 randomized or observational studies were included in the final review. Digital interventions most commonly used mainstream, existing social media platforms (n=7) or promotion through online peer educators (n=5). Most interventions (n=8) were categorized as interactive and allowed user engagement and most directly facilitated testing (n=7) either by providing self-testing kits or referral to testing services. A total of 1930 participants were included across the 13 studies. HIV testing uptake among MSM and TW exposed to digital interventions was 1.5 times higher than that of unexposed MSM and TW (risk ratio [RR] 1.5; 95% CI 1.3-1.7). Subanalyses suggested an increased impact on HIV testing uptake among interventions that were delivered through mainstream social media–based platforms (RR 1.7; 95% CI 1.3-2.1), included direct facilitation of HIV testing (RR 1.6; 95% CI 1.4-1.9), were interactive (RR 1.6; 95% CI 1.4-1.8), and involved end users in the design process (RR 1.6; 95% CI 1.3-2.0).

**Conclusions:**

These findings provide broad support for the integration of technology with existing approaches to promote and facilitate HIV testing among MSM and TW. Our findings identified key features that may be associated with greater impact on HIV testing uptake and can be used to inform future development efforts given the growing interest and application of digital technologies in HIV prevention.

**Trial Registration:**

PROSPERO International Prospective Register of Systematic Reviews CRD42017070055; https://www.crd.york.ac.uk/prospero/display_record.php?ID=CRD42017070055.

## Introduction

Globally, men who have sex with men (MSM) and transgender women (TW) are disproportionately affected by HIV [1-4]. Evidence of expanding epidemics among MSM have been noted, with new infections among this group comprising up to half of all incident cases in some regions, including North America [5-9], and up to approximately 70% in specific countries, such as the Philippines [7]. This burden of HIV occurs against a background of expanded access to HIV testing and treatment and the emergence and growing coverage of biomedical HIV prevention strategies [5].

The timely diagnosis of HIV plays an important role in preventing transmission, both by prompting reductions in risk behaviors to prevent onward transmission [10,11] and by facilitating early access to treatment and viral suppression. It is now known that people with suppressed HIV cannot transmit the virus to others [12-15]; promoting regular HIV testing is therefore a key global prevention strategy [16]. However, a range of barriers faced by MSM and TW limits their access to HIV testing and other preventive services. Stigma and discrimination remains a key deterrent to HIV service utilization in many parts of the world, particularly in settings where legislation prohibits same-sex relationships and legitimizes discriminatory behavior toward sexual minorities [17-19]. Other structural barriers, such as accessibility of services, costs, waiting time, and confidentiality concerns [20-24], also limit access to HIV testing among MSM and TW and prevent levels of testing coverage required to impact HIV incidence [25].

The use of digital communication technology—internet-enabled technology such as mobile phones, computers, and tablets that allow access to digital platforms and apps—to promote HIV testing among MSM and TW is an area of growing interest. Digital technology has assumed a prominent role in contemporary gay culture [26], and to a lesser extent in TW communities [27], as a tool to meet sex partners [28-30]. Evidence pointing to higher rates of condomless sex [31-33] and diagnoses of sexually transmitted infections (STI) [27,28,34,35] among MSM and TW who use online platforms to find sex partners suggests that online platforms may be appropriate targets for sexual health promotion. It has been suggested that internet-enabled technology can provide a discrete means for HIV health promotion in locations where same-sex behaviors are highly stigmatized or illegal [36], while the acceptability of HIV prevention interventions delivered through digital communication technologies has been noted among MSM and TW [37-43]. Together, these findings enhance the potential of using digital technology to reach those at high risk of HIV and serve as an important platform for health promotion outreach.

We performed a systematic review and meta-analysis to describe the impact of digital communication technology interventions on the uptake of HIV testing among MSM and TW. Subanalyses were undertaken to identify intervention characteristics associated with increased impact.

## Methods

### Overview

We systematically searched for current literature that describes the impact of digital communication technology interventions on the uptake of HIV testing among MSM and TW. We defined digital communication technology as technologies that were internet-enabled (through devices such as computers, mobile phones, or tablets) and which provided access to digital platforms such as social media sites, websites, apps, and email. The systematic review was conducted in accordance with the Preferred Reporting for Systematic Reviews and Meta-Analyses guidelines [44] and was registered on PROSPERO (registration number CRD42017070055).

Eligible studies were defined as those that:

1. Utilized at least one digital communication platform to deliver an intervention to promote HIV testing
2. Reported uptake of HIV testing as a result of a digital communication intervention for MSM and TW participants
3. Measured impact either by prospectively comparing testing rates pre- and postintervention exposure within a single cohort or through a randomized study design.

Studies that measured HIV testing outcomes but utilized digital communication technologies primarily for a purpose not directly related to improving HIV testing uptake (eg, to facilitate data collection or recruitment) and those reporting intention to test outcomes only were excluded.

Uptake of HIV testing was defined as any quantitative count of HIV testing events among MSM and TW measured using either self-report or clinic records.

For randomized controlled studies, we report on HIV testing uptake among participants in the intervention and control groups at the study endpoint. For nonrandomized studies, we report on HIV testing uptake at pre-intervention baseline and postintervention study endpoints.

### Search Strategy

We conducted a systematic search of the literature published in English using the Medical Literature Analysis and Retrieval System Online, or MEDLARS Online (MEDLINE), Cumulative Index of Nursing and Allied Health Literature (CINHAL), EMBASE, PubMed, and PsychInfo databases. We limited our search to studies published between January 1, 2010, and May 1, 2018, to account for the redundancy of older platforms and technologies. Our search strategy comprised key terms; Medical Subject Headings (MeSH) terms; and subject headings related to participant (eg, *MSM*), intervention (eg, *internet*), and outcome (eg, *HIV testing and counseling*) variables (Multimedia Appendix 1 shows the illustration of search strategy and MeSH terms). Electronic repositories of the International AIDS Society and the International AIDS Conferences were manually searched for abstracts from January 2010 onward. Reference sections of the identified papers were also searched for additional papers. No restrictions were placed on participant age or other demographic characteristics of the study population or geographical location or setting of the intervention.

### Data Extraction

Following the literature search, the first author (VV) removed duplicate records and assessed the remaining abstracts on the basis of the eligibility criteria. The second author (KR) reviewed a random sample (equivalent to 10%) of discarded abstracts to ensure accuracy. Both authors then conducted a full-text review of the remaining abstracts to determine their inclusion in the final analysis.

The following domains were extracted for final analysis by two authors (VV and KR) using a standardized, Excel-based tool: study identification, study design (data collection period, recruitment, and sampling method), intervention characteristics (aim of intervention, mode of intervention delivery, and duration), study population (inclusion and exclusion criteria, sample size, primary HIV testing outcome used and time frame for the outcome, the mean age of sample, proportion reporting previous testing), and results and analysis (number of testing outcomes, effect size measurement, and reported effect size).

Any discrepancies identified between the two authors during the review and data extraction process were discussed with a third author (MS).

### Analysis

#### Qualitative Synthesis of Study Aims, Intervention Characteristics, and Study Outcomes

A qualitative synthesis was undertaken to characterize included studies by their study participants, location of study, study design, digital communication intervention platform, intervention features, length and frequency of exposure, sample size, and rated study quality. For each study, we then described the proportion of MSM and TW reporting previous HIV testing at baseline, how testing uptake was defined and measured during the study period, whether testing was provided or offered as part of the intervention, and the proportion of MSM and TW reporting or receiving HIV testing at the end of the study period.

The primary outcome of this study was uptake of HIV testing among MSM and TW participants, which we defined as the number of individual MSM and TW reporting or receiving an HIV test divided by the total number of MSM and TW exposed to a digital intervention. Intervention effectiveness was determined by manually calculating risk ratios (RRs) comparing testing uptake between exposed groups and unexposed groups for randomized controlled trials (RCTs) or between pre- and postintervention commencement for nonrandomized studies. HIV testing uptake among controls or at preintervention time point was used as the reference group so that an RR greater than one demonstrates a higher chance of HIV testing uptake following exposure to a digital communication intervention.

#### Meta-Analysis

We performed a meta-analysis of intervention effectiveness to generate pooled RR using a DerSimonian and Laird random effects model to account for the anticipated heterogeneity between studies. We identified a range of characteristics common to digital communication interventions or regarded as features that potentially enhance the intervention effectiveness and performed submeta-analyses to generate pooled RR to examine the impact of these characteristics on overall intervention effectiveness:

1. Intervention interactivity (yes/no): interventions that permitted end users to interact or engage, for example, by chatting with peer educators or other participants, as opposed to passive viewing of an online video
2. End-user involvement in the design process (yes, no, or not reported): any reported involvement of the intended end users in the intervention design process (eg, consultation and pilot testing)
3. HIV testing facilitated as part of the intervention (yes/no): interventions that directly provided (eg, provided self-tests) or facilitated (eg, direct referral) HIV testing to participants, as opposed to simply promoting HIV testing
4. Social media platform (yes/no): interventions implemented through an established social media platform (eg, Facebook) that facilitates social networking among the general population or specifically among gay and other MSM, as opposed to nonsocial media platforms
5. Single dose exposure (yes/no): interventions that delivered a single, time-bound exposure as opposed to multiple exposures over time.

The presence and magnitude of heterogeneity were assessed in meta-analyses using the χ^2^ and I^2^ tests, respectively. All statistical analyses were performed using Stata version 14 (StataCorp LP).

### Assessing Study Quality

Study quality was assessed by two authors (VV and KR) using the quality assessment tool for quantitative studies [45]. This tool critiqued studies on the basis of selection bias, study design, confounders, blinding, data collection methods, and withdrawal and dropouts. Each criterion was rated as strong, moderate, or weak. On the basis of the combined scores, studies were given a final, global rating of strong (no weak ratings), moderate (one weak rating), or weak (two or more weak ratings). As the final global rating was determined by the number of subcategories scored as *weak*, the tool was modified to include an *N/A* option for blinding and withdrawal and dropout fields to account for nonrandomized studies. The first and second authors (VV and KR) met after completing the first two quality assessments to ensure consistency in the use of the tools. After consistency was confirmed, the two authors completed the remaining quality assessments, and the final results were compared and discussed. Similar to the process for data extraction, any disagreements between the assessments of the first and second author were discussed with a third senior author (MS).

## Results

### Search Results

**Figure 1:**
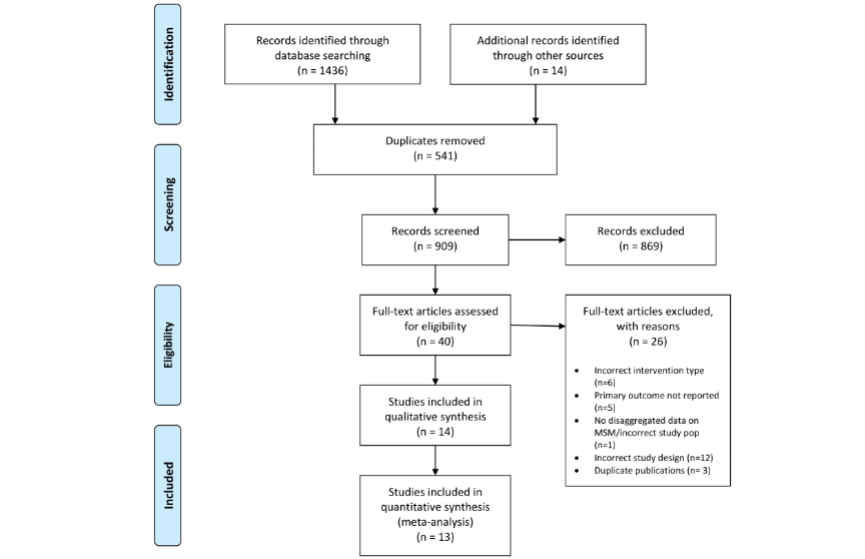
The Preferred Reporting Items for Systematic Review and Meta-Analyses (PRISMA) flow diagram depicting study screening and selection.

The systematic literature search resulted in 1436 identified records, including 8 conference abstracts and 6 papers identified in the references of the included studies. 37.30% (541/1436) were removed as duplicates. The remaining 909 records were reviewed at the abstract level, 4.4% (40/909) of which were retained for full-text review. Thirteen papers were included in the final analysis [46-58] (Figure 1).

### Study and Sample Characteristics

Table 1 presents the characteristics of the included studies. All included studies specifically targeted men who identified as gay or self-reported anal sex with a male partner. Two studies included TW [53,54]. Study participants were typically aged in their midtwenties, and the overall level of educational attainment varied substantially across studies. On the basis of the World Bank classifications [59], the majority of studies took place in high-income countries (Hong Kong, n=1; Taiwan, n=1; and United States, n=6), 4 occurred in upper-middle-income countries (China, n=1 and Peru, n=3), and 1 in a low-middle-income country (India). The studies collectively included 8875 participants, 64 (0.70%) of whom were reported as TW, with a mean sample size of 682 participants (SD 808; range 56-3092 participants). Two studies excluded participants with a history of HIV testing, and two studies did not report on participants’ previous testing history. Among the 9 remaining studies, the mean proportion of participants reporting a history of HIV testing was 44.1% (SD 19.0; range 21.3%-73.8%). The recall period for these studies varied, with one study reporting lifetime HIV testing history and the remaining studies using recall periods that ranged from 3 months to 3 years. A total of 10 studies used an RCT design [46-49,51,54-58]. The specific study design used by the remaining 3 studies was a prospective cross-sectional study with nonequivalent control group [50], a prospective single-group cross-sectional study [52], and a prospective cross-sectional matched-pair randomized trial design [53]. Due to the lack of individual-level randomization and nonequivalence of control groups, we calculated relative risk for these 3 studies using reported HIV testing uptake at preintervention baseline and postintervention endpoints in the intervention arm only. One RCT described two intervention arms and did not present disaggregated findings; therefore, this study was treated as a single cohort, and relative risk was calculated based on HIV testing uptake at baseline and endpoint [51].

### Aims of Included Studies

Promoting HIV testing uptake was identified as the primary aim of all 13 studies. Five studies had multiple primary aims: mutual disclosure of HIV status with sexual partners (ie, asking and disclosing HIV serostatus [49]), STI testing [46], intention to test [47,51], and reduction of unprotected anal intercourse [49,50] (data not reported).

**Table 1:** Overview of included studies.

Reference	Description of study participants	Location (country of income classification^a^)	Total sample size	TW^b^ participants, n (%)	Percentage of participants reporting previous HIV testing preintervention (time frame for testing history)	Study design
Bauermeister et al (2015) [46]	Inclusion criteria: Young MSM^c^ aged 15-24 years, self-identified cis male, reported sex with a male partner in the past 6 months; sample characteristics: mean age 21 years, 92.3% educated to high school or GED^e^ level	Michigan, United States (high)	130	0 (0)	73.8 (lifetime)	RCT^d^
Blas et al (2010) [47]	Inclusion criteria: MSM aged 18 years and above, reporting lifetime sex with another man, and not reporting testing within the past 12 months; sample characteristics: mean age 26.1 years (range 18-61), 42% educated to university or technical graduate level	Lima, Peru (upper middle)	459	0 (0)	21.3 (more than 1 year ago)	RCT
Blas et al (2014) [48]	NR^f^	Peru (upper middle)	400	0 (0)	NR	RCT
Hirshfield et al (2012) [49]	Inclusion criteria: male aged 18 years or above, reporting oral or anal sex with a current male partner and oral, anal, or vaginal sex with at least one new partner (male or female) in the past 6 months; sample characteristics: median age 39 years (range 18-81), 55% educated to college degree or higher level	United States (high)	3092	0 (0)	69.0 (past 3 years)	RCT
Ko et al (2013) [50]	Inclusion criteria: MSM aged 18 years and above, reported sex with another man in the past 12 months; sample characteristics: mean age 24.8 years, 63% educated to college level	Taiwan (high)	1037	0 (0)	29.4 (past 6 months)	Prospective cross-sectional study with nonequivalent control
Patel (2016) [51]	Inclusion criteria: MSM aged 18 years or older living in Mumbai	Mumbai, India (low middle)	244	0 (0)	61.5 (past 6 months)	RCT
Rhodes et al (2011) [52]	Inclusion criteria: male, registered user of chat room servicing MSM in North Carolina, United States; mean age 37 years; gay-identifying; sample characteristics: mean age 37.1 years (range 18-71 years; education not reported)	North Carolina, United States (high)	346	0 (0)	44.5 (past 3 years)	Prospective cross-sectional study
Rhodes et al (2016) [53]	Inclusion criteria: male, social media user, mean age 40 years, gay-identifying; sample characteristics: mean age 40.9 years (range 18-74 years; education not reported)	United States (high)	1292	28 (2.17)	36.6 (past 12 months)	Prospective cross-sectional matched-pair randomized trial design
Tang et al (2016) [54]	Inclusion criteria: male aged 16 years or above, reporting lifetime anal sex with another man and no HIV testing history; sample characteristics: 37% aged between 21 and 25 years, 65% college educated	China (upper middle)	721	36 (5.0)	N/A^g^	RCT
Washington et al (2017) [55]	Inclusion criteria: Black or African American male aged 18-30 years reporting sex with another man during the past 3 months and not tested within the past 6 months; sample characteristics: mean age 23.1 years, 52% educated to high school or GED level	Los Angeles, United States (high)	56	0 (0)	46.5 (past 12 months)	RCT
Wang et al (2018) [56]	Inclusion criteria: Chinese-speaking males in Hong Kong aged 18 years or over reporting anal sex with 1 or more male partner in the past 6 months with access to online live chat apps (Line, WhatsApp, and Skype); sample characteristics: 63.1% aged between 18 and 31 years, 81.4% educated to a university level	Hong Kong, China (high)	430	0 (0)	N/A	RCT
Young et al (2013) [57]	Inclusion criteria: African American or Latino male aged 18 years or above, registered Facebook users, reporting sex with another man in the past 12 months; sample characteristics: mean age 31.8 years (SD 10.2 years), 36.4% educated to high school level	Los Angeles, United States (high)	112	0 (0)	NR	RCT
Young et al (2015) [58]	Inclusion criteria: MSM aged 18 years and over, reporting sex with another man during the past 12 months; sample characteristics: 28.9 mean age (SD 7.9 years), 37.8% educated to vocational school level	Lima, Peru (upper middle)	556	0 (0)	33.4 (past 3 months)	RCT

^a^Classification based on World Bank countries and lending groups [59].

^b^TW: transgender women.

^c^MSM: men who have sex with men.

^d^RCT: randomized controlled trial.

^e^GED: general education development.

^f^NR: not reported.

^g^N/A: not applicable.

### Intervention Characteristics of the Included Studies

Table 2 presents the characteristics of the digital interventions among the included studies. Studies utilized a variety of digital communication platforms to deliver interventions. Six used only social media platforms, 3 used only online videos, and 1 delivered a tailored online HIV/STI testing intervention through a customized website. The remaining 3 studies used multiple platforms: 1 used online videos in conjunction with motivational messages sent via email or instant messaging, 1 used social media and live chat apps, and 1 used online videos in conjunction with live chat apps.

Of the 7 studies that used social media, 5 delivered interventions using Facebook [50,51,55,57,58]. All of these studies used closed or private Facebook groups to promote HIV testing, using a range of accompanying features and modalities using internet popular opinion leaders to disseminate HIV-related information and engage in conversations about HIV testing, prevention, and risk behavior [50]; using trained peer educators to deliver HIV prevention and testing information and promote HIV testing uptake [55,58], including through the provision of HIV self-tests [57]; delivering weekly videos promoting HIV testing alongside moderated group discussion [47]; and sending HIV prevention and HIV testing promotion messages (in conjunction with messages sent by WhatsApp and email) [51].

Two social-media-based interventions were delivered through sites specifically targeting the gay community [52,53]. Both reported on the Cyber-Based Education and Referral/testing (CyBER/testing) intervention, which utilized trained peers to promote HIV testing through existing social and sexual networking sites popular among the gay community. CyBER/testing was implemented through an existing chat room used by MSM [52] and later through four geographically focused social media sites used by MSM and TW [53], both in the United States.

Only one intervention was delivered through a custom-built website (Get Connected!), which aimed to promote and connect HIV and STI testing to young MSM [46]. The website delivered customized content to participants based on sociodemographic, sexual identity, and behavior and previous engagement with HIV testing data provided during a baseline assessment, so that messaging and content were personalized to mirror participant profiles, and experiences with testing, including motivations and perceived barriers.

Four studies used online videos to promote HIV testing [47-49,54]. One used single-session online videos focusing on HIV prevention to motivate HIV testing among MSM in the United States, in which participants received either dramatic- or documentary-style videos, or both [49]. A 2010 intervention delivered one 5 min video promoting HIV testing customized based on self-reported sexual identity (gay or nongay identified) to MSM in Peru [47]. In 2014, this intervention was repeated with the addition of motivational messages sent by email or instant messaging to encourage HIV testing to MSM in Peru [48]. Finally, online videos developed with crowdsourced content depicted two Chinese men falling in love and getting tested together to target MSM and TW naïve testers in China [54]. One study used online videos in combination with live chat apps to deliver a home-based HIV self-testing service to MSM in Hong Kong [56]. In this study, all participants were exposed to an online video promoting HIV testing, and then, the participants in the intervention group were offered a home-based self-testing kit and online, real-time HIV pre- and posttest counseling and instruction through Line, WhatsApp, or Skype.

Regarding the length of intervention and the frequency of exposure, interventions that used online videos were typically shorter in duration and involved a single exposure to a video [47,54,56]. Two studies sent multiple videos [49,55] and one combined videos and motivational messages via text, email, or instant messaging but did not report on the length or number of videos and frequency of motivational messages [48]. The customized website-based intervention [46] also provided a single exposure to the website content, but data on the length of time spent on the website were not reported.

Most interventions (n=8) were categorized as interactive for their ability to allow user engagement, for example, through social media platforms, responses on messaging platforms or through email, or via a website. Interventions involving online peer educators interacting with participants through social media platforms were typically longer in duration, ranging from 12 weeks [51,57,58] to 12 months [53]. The frequency of exposure to online peer educators was user-determined, that is, participants were free to choose how often, if at all, they interacted with online peer educators. The exception involved participants receiving twice weekly messages through Facebook, email, or instant messaging, over a 12-week period [51].

Seven studies directly facilitated HIV testing, through the provision of HIV home or self-testing kits [56,57]; referrals to specific, local HIV testing clinics [47,48,58]; or providing location details of free, local HIV testing sites [51,55]. The remaining six studies provided general promotion of HIV testing only.

Five studies specifically mentioned the involvement of intended end users in the implementation design process [46,47,52,55,56]. Theoretical underpinning to intervention development was described by 9 studies [46,47,49,51-53,55,56,58] (Table 2).

**Table 2:** Characteristics of digital interventions of included studies.

Reference	Platform	Description	Interactive	Intervention length (frequency of exposure)	HIV testing provided or facilitated	Measurement of HIV testing uptake used	Length of follow-up period for outcome	Comparator	Involvement of end users in intervention design	Theoretical framework
Bauermeister et al (2015) [46]	Website	Interactive, customized website (Get Connected!) that delivered HIV/STI^a^ testing and prevention content tailored to specific participant profiles of based on psychosocial data and previous engagement with HIV testing	Yes	One time	No	Self-reported	30 days	Control group: test-locator website	Yes	Self-determination theory principles and integrated behavioral model
Blas et al (2010) [47]	Online video	One 5-min video delivered through existing gay and commercial websites promoting HIV testing customized based on self-identification of participant as either gay or nongay	No	One time (5 min)	Yes—facilitated (referral)	Attendance based	125 days^b^	Control group: standard public health text	Yes	Health belief model
Blas et al (2014) [48]	Multiple: online videos, email/instant messaging	Motivational videos and messages about HIV testing sent through email and instant messaging, respectively	No	NR^c^	Yes—facilitated (referral)	Attendance based	184 days^b^	Control group: health promotion message with invitation for free HIV testing	No or not reported	None reported
Hirshfield et al (2012) [49]	Online video	HIV prevention videos in either dramatic or documentary style (or both), accessed via banner ads on gay-oriented sexual networking sites, and designed to promote critical thinking about HIV disclosure, testing, and condom use	No	One time (9 and 5 min)	No	Self-reported	60 days	Control group: no content	No or not reported	Social learning theory
Ko et al (2013) [50]	Social media (Facebook)	Trained internet popular opinion leaders promoting HIV testing and prevention to members of a closed Facebook group	Yes	6 months (user-dependent)	No	Self-reported	6 months	Baseline	No or not reported	None reported
Patel (2016) [51]	Multiple: social media (Facebook); online live chat apps (WhatsApp); email	16 health promotion messages promoting HIV testing framed in either approach or avoidance style of messaging sent by trained peers via their preferred modality (private Facebook group, individual WhatsApp messaging, or email)	No	12 weeks (twice weekly)	Yes—facilitated (test locator)	Self-reported	12 weeks	Baseline	No or not reported	Information motivation behavioral skills model
Rhodes et al (2011) [52]	Social media (MSM^d^-specific sites)	Trained peer posting regular triggers about HIV and HIV testing in existing chat room used by gay and other MSM and engaging in direct communication about testing services, processes, and locations with chat room users	Yes	6 months (daily)	No	Self-reported	6 months	Baseline	Yes	Natural helping
Rhodes et al (2016) [53]	Social media (MSM-specific sites)	Trained peer posting regular triggers in four existing social media sites used by gay and other MSM about HIV and HIV testing and engaging in direct communication with users about testing services, processes, and locations	Yes	12 months (daily)	No	Self-reported	12 months	Baseline	No or not reported	Empowerment education, social cognitive theory, and natural helping
Tang et al (2016) [54]	Online video	Online video promoting HIV testing based on a crowdsourced design accessed via banner ads placed on gay-oriented social networking platforms	No	4 weeks (one time)	No	Self-reported	3 weeks	Control group: noncrowd sourced online video (standard public health text)	Yes	None reported
Washington et al (2017) [55]	Social media (Facebook)	Five, 1-min long videos promoting HIV testing sent through a private Facebook group to black or African American MSM, with moderated group discussion	Yes	6 weeks (weekly)	Yes—facilitated (test locator)	Self-reported	6 weeks	Control group: closed Facebook group receiving generic health information	Yes	Integrative model of behavior change
Wang et al (2018) [56]	Multiple: online videos; online live chat apps (Line, WhatsApp, and Skype)	Home-based self-testing service comprising online promotional video about HIV testing, plus additional videos on home-based HIV self-testing and offer of free HIV self-testing kit and online real-time instructions and pre- and posttest counseling provided via live chat apps	Yes	6 months (one time)	Yes—provided (HIV self-testing)	Self-reported or observed uptake of self-testing	6 months	Control group: online video about (general) HIV testing only	Yes	Health belief model
Young et al (2013) [57]	Social media (Facebook)	Trained peer educators providing HIV prevention and testing messages, including 4 weekly reminders about availability of HIV home testing, to participants of a closed Facebook group	Yes	12 weeks (user -dependent)	Yes—provided (HIV self-testing)	Requested and returned home-based HIV testing kit and followed-up results	12 weeks	Control group: closed Facebook group receiving per-delivered generic health information	No or not reported	None reported
Young et al (2015) [58]	Social media (Facebook)	Trained peer educators providing HIV prevention and testing messages, including 4 weekly reminders about availability of HIV home testing, to participants of a closed Facebook group	Yes	12 weeks (user-dependent)	Yes—facilitated (referral)	Attendance based	12 weeks	Control group: closed Facebook group providing HIV testing information without peer leaders	No or not reported	Diffusions of innovation theory and social normative theory

^a^STI: sexually transmitted infection.

^b^Reported as the average follow-up time.

^c^NR: not reported.

^d^MSM: men who have sex with men.

### Outcomes of the Included Studies

Table 3 presents the reported outcomes of studies using the RCT study design. Most RCT studies measured testing uptake through self-reports [46,49,54,55]. The length of follow-up over which testing was measured varied greatly from 3 weeks [54] to 6 months [56].

On the basis of the calculated RRs, 3 of 9 RCTs demonstrated a significant improvement in HIV testing uptake [55,56,58], whereas 4 demonstrated nonsignificant improvements [46,47,49,54]. The two remaining RCTs did not demonstrate any impact; in one RCT, testing uptake was extremely low in both intervention and control arms [48], and in another, no members of the control group tested for HIV during follow-up [57].

**Table 3:** Reported HIV testing outcomes in included randomized controlled trial studies.

Reference	Control group		Intervention group		Risk ratio (95% CI)	
	Total number of participants	Participants tested, n (%)	Total number of participants	Participants tested, n (%)		
Bauermeister et al (2015) [46]	36	4 (11)	68	18 (26)		2.4 (0.9-6.5)
Blas et al (2010) [47]	220	10 (4.5)	239	19 (7.9)		1.7 (0.8-3.7)
Blas et al (2014) [48]	200	3 (1.5)	200	2 (1.0)		0.7 (0.1-3.9)
Hirshfield et al (2012) [49]	240	48 (20.0)	676	142 (21.0)		1.1 (0.8-1.4)
Tang et al (2016) [54]	317	111 (35.0)	307	114 (37.1)		1.1 (0.9-1.3)
Wang et al (2018) [56]	215	109 (50.7)	215	193 (89.8)		1.8 (1.5-2.0)
Washington et al (2017) [55]	22	8 (36)	20	16 (80)		2.2 (1.2-4.0)
Young et al (2013) [57]	55	0 (0)	57	8 (14)		N/A^a^
Young et al (2015) [58]	246	16 (6.5)	252	43 (17.1)		2.6 (1.5-4.5)

^a^N/A: not applicable.

Table 4 presents the outcomes from nonrandomized studies. All of the 4 nonrandomized studies relied on self-reported HIV testing uptake. The length of follow-up ranged from 12 weeks [51] to 12 months [53], and all 4 studies demonstrated significant improvements in testing uptake based on calculated RRs (Table 4).

Across all 13 studies included in this review, 21.75% participants (1930/8875) received an HIV test during a cumulative 3.6 years of study follow-up. Three studies reported on HIV diagnoses (all RCTs) [47,54,56], with 75 new HIV infections detected across these studies (56% in the intervention arm; data not reported).

**Table 4:** Reported HIV testing outcomes in included quasi-experimental studies.

Reference	Baseline		End line		Risk ratio (95% CI)
	Total number of participants	Participants tested, n (%)	Total number of participants	Participants tested, n (%)	
Ko et al (2013) [50]	501	150 (29.9)	499	219 (43.9)	1.5 (1.2-1.7)
Patel (2016) [51]	130	42 (32.3)	130	57 (43.8)	1.4 (1.0-1.9)
Rhodes et al (2011) [52]	346	154 (44.5)	315	187 (59.4)	1.3 (1.4-1.5)
Rhodes et al (2016) [53]	353	122 (34.6)	399	216 (54.1)	1.6 (1.3-1.9)

### Meta-Analysis

#### Primary Outcome

**Figure 2:**
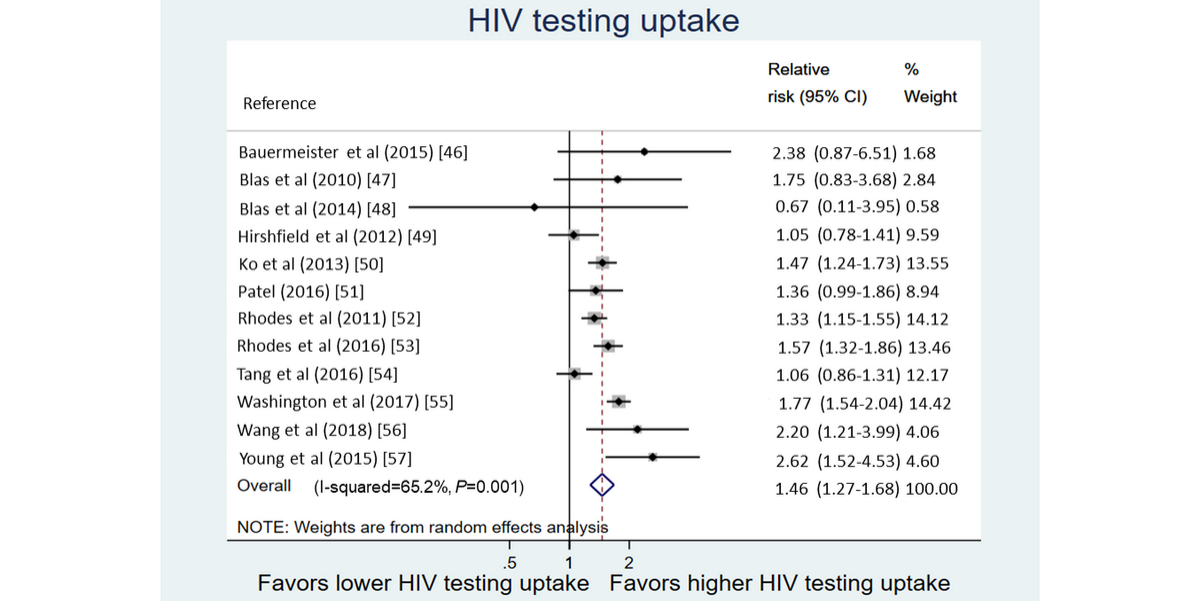
Forest plot of relative risk of HIV testing uptake among MSM and TW following digital intervention exposure.

The pooled RR across 12 studies (RR could not be calculated for one study because no tests were recorded in the control arm) [57] indicated a significant increase in the uptake of HIV testing following exposure to digital interventions (RR 1.5; 95% CI 1.3-1.7). Statistical heterogeneity was high (χ^2^_11_=31.7; I^2^=65.2%; Figure 2).

#### Subanalysis of the Study Characteristics

A positive impact was seen on the HIV testing uptake across all intervention type subanalyses. The highest pooled RR was seen for interventions that were delivered through the mainstream social media–based platforms (RR 1.7; 95% CI 1.3-2.1), interventions that included direct facilitation of HIV testing (RR 1.6; 95% CI 1.4-1.9), interventions that were interactive (RR 1.6; 95% CI 1.4-1.8), and interventions that involved end users in the design process (RR 1.6; 95% CI 1.3-2.0; Table 5).

**Table 5:** Subanalyses by selected study and intervention characteristics.

Intervention characteristic		k^a^	Risk ratio (95% CI)	χ^2^ (*df*)	I^2^
Overall effect size		12	1.5 (1.3-1.7)	31.7 (11)	65.2
Randomized controlled trials only		8	1.6 (1.2-2.1)	29.1 (7)	76.0
Quasi-experimental		4	1.4 (1.3-1.6)	2.1 (3)	0
**Direct facilitation of HIV testing**					
	Yes	7	1.6 (1.4-1.9)	13.8 (6)	65.2
	No	5	1.3 (1.0-1.6)	9.4 (4)	58.1
**Interactive intervention**					
	Yes	7	1.6 (1.4-1.8)	15.0 (6)	46.7
	No	5	1.1 (1.0-1.3)	1.7 (4)	65.2
**User involvement in design**					
	Yes	5	1.6 (1.3-2.0)	9.4 (4)	57.6
	No or not reported	6	1.4 (1.1-1.6)	18.0 (5)	66.7
**Theoretical basis to intervention**					
	Yes	9	1.6 (1.3-1.8)	20.5 (8)	61.0
	No or not reported	3	1.2 (0.9-1.7)	6.2 (2)	67.5
**Social media–based intervention**					
	Yes—general	4	1.7 (1.3-2.1)	6.0 (3)	49.8
	Yes—gay oriented	2	1.4 (1.2-1.7)	2.0 (1)	48.7
	No	6	1.4 (1.0-1.9)	23.1 (5)	78.4
**Single-dose intervention**					
	Yes	5	1.4 (1.0-1.9)	22.4 (4)	82.1
	No	6	1.5 (1.3-1.7)	8.5 (5)	41.2

**^a^**k: number of studies included in the subcategory.

### Study Quality

The majority of studies (n=8) were classified as *moderate* quality [46,47,52-56,58], 4 were classified as *weak* [48-51], and 1 was classified as *strong* [57]. The most common limitation across studies was insufficient description of blinding procedures (rated as *weak* in 9 studies), whereas controlling for confounding in either study design or analyses was a common strength (rated as *strong* in 9 studies; Multimedia Appendix 2).

**Figure 3:**
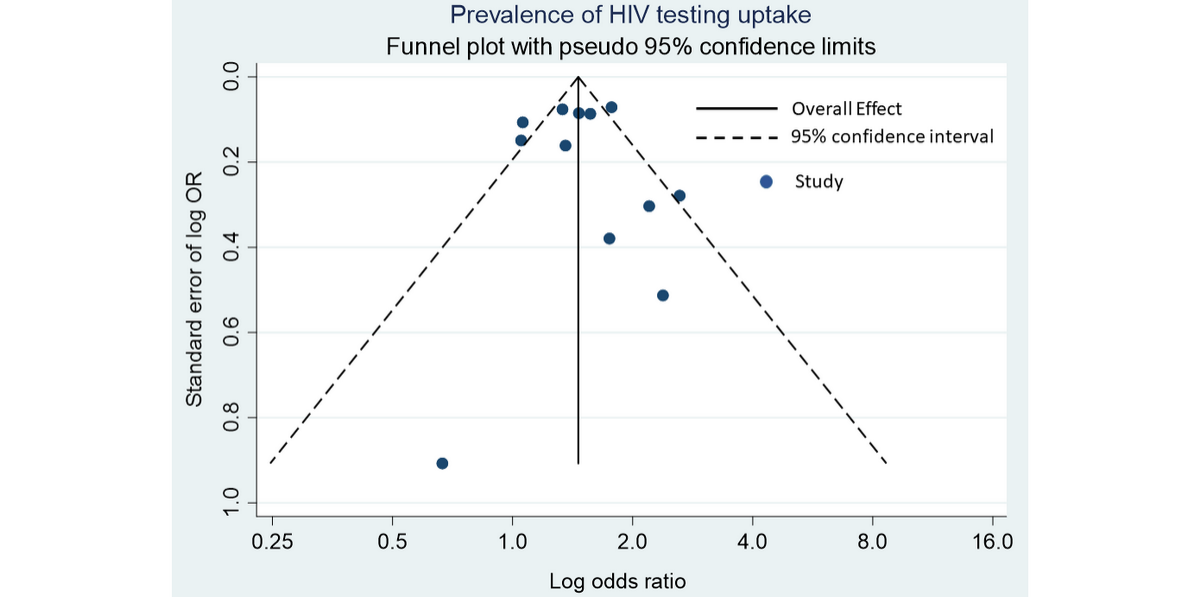
Funnel plot for estimating publication bias and precision of estimate.

The observed asymmetry in the study funnel plot (Figure 3) may be explained by the heterogeneity between studies, given the variability in the intervention design. The attribution of asymmetry to heterogeneity is also supported by the high level of variance between studies (I^2^=65.2%).

## Discussion

In this systematic review, exposure to digital communication interventions was associated with greater HIV testing uptake among MSM and TW compared with those unexposed to digital communication interventions. Our findings provide broad support for the integration of technology into existing tools and approaches to HIV prevention among MSM and TW. We extend the current state of evidence regarding the impact of digital interventions on HIV testing by examining the role of key intervention features on effectiveness. Our findings identified key features that were associated with greater uptake for HIV testing for MSM and TW—specifically, interventions that facilitate HIV testing through provision or direct referral to HIV testing services, the use of mainstream social media platforms to engage the target population, interactivity, and involvement of end users in design processes.

Global guidance has recommended the integration of digital technology as a strategy to enhance the reach and effectiveness of HIV prevention efforts among MSM and TW [36,60]. Numerous acceptability studies have identified that MSM would be willing to use phone- and web-based technologies for HIV prevention [39,40,61,62]. Recent systematic reviews have explored the potential of digital technology to advance various HIV priorities among MSM and other key populations [63,64]. One systematic review to date has attempted to quantify the role of digital communication technologies in increasing engagement among key populations across the care continuum through meta-analysis [65]; this study specifically looked at randomized and observational studies describing the impact of social media–based interventions on HIV testing uptake among key populations. All 9 studies included in this meta-analysis targeted MSM and were collectively associated with an approximately 50% increase in HIV testing uptake. Compared with this review, our study adopted a broader definition of digital interventions that encompassed websites, online videos, instant messaging, and live chat apps, resulting in the inclusion of 6 additional studies. Overall, we found that exposure to digital interventions was associated with an approximately 50% increase in HIV testing uptake, in line with the previous study’s estimates, supporting the conclusion that digital communication technologies are effective in promoting HIV testing among MSM and TW. Interventions included in this review invariably took advantage of various online engagement tools such as videos, digital messages tailored to participant profiles, and online social networking and compared the outcomes with more generic and less interactive online messaging. The impact of digital communication technology to increase testing uptake may be even greater when compared with more traditional health promotion mediums such as messaging through public media (such as posters and billboards). In addition, our study identified specific features of digital interventions associated with greater impact on HIV testing uptake among MSM and TW.

First, interventions that went beyond creating general demand for testing, either through health promotion or providing educational content, and instead directly facilitated HIV testing through service referrals or the provision of self-tests demonstrated greater impact on HIV testing uptake compared with the overall estimate. Direct facilitation of HIV testing may potentially address some of the more structural barriers MSM and TW face to HIV testing [17,66,67]. In particular, HIV self-testing has emerged as a strategy to mitigate barriers related to HIV service access [68,69]; however, some MSM and TW populations have expressed concerns about the limited availability of support during the testing process [70,71]. The study by Wang et al [56] included in this review suggests that digital interventions can play a role in promoting HIV self-testing by mitigating user concerns through the provision of real-time, online counseling. It should also be noted that the majority of studies included in this review recruited participants with generally high levels of previous HIV testing behaviors, which may be critical to the success of this approach. Past research has shown that a history of testing is a strong predictor of future testing behaviors among MSM [72-74], suggesting that the direct facilitation of testing through digital communication interventions may work best for those who are experienced in HIV testing and may enable participants to access more frequent testing.

Second, interactive interventions—those that allowed participants to engage directly with online content or other users—demonstrated greater impact on HIV testing uptake compared with the overall pooled estimate. Interactivity in digital interventions has been associated with achieving a greater impact on behavior change across a range of health areas [75-77] and has been identified as a desired feature of digital HIV prevention interventions among MSM and TW [43,78]. Third, our subanalysis also identified that interventions that used mainstream social media platforms, such as Facebook, were also associated with greater uptake of HIV testing. However, all interventions that utilized social media platforms were also categorized as interactive, making it difficult to isolate the source of the enhanced effect. Social networking–based interventions are commonly used for sexual health promotion [79], and the use of existing social media platforms has been identified as a way to enhance retention among young MSM and TW in online HIV prevention activities [43]. Using existing and well-utilized social media platforms may also enhance the reach of digital HIV prevention interventions [80] compared with those that are delivered through new or separate platforms. Interestingly, interventions that used social media sites specifically for gay and other MSM were less effective in increasing HIV testing uptake than interventions delivered through general social media. Others have noted the reluctance of users of gay social networking sites to receive health promotion messages, which are often seen as an intrusion or surveillance and may limit user engagement [81].

Fourth, evidence of enhanced impact was found among digital interventions that reported involvement of end users in the design process. This involvement is a key component of user-centered design, an approach that prioritizes user needs and experiences to maximize functionality and increase engagement and relevance to the target population [82-84]. User involvement is particularly important when developing digital interventions tailored to specific target populations to ensure that such interventions appropriately reflect group priorities, preferences, and culture [83]. Although the literature confirms the value of user-centered design, our review only assessed studies on whether any involvement of end users in the design process was reported; however, it is probable that the quality and depth of this involvement may be a stronger determinant of overall effectiveness. In addition, grounding in theoretical frameworks may also be another indicator of effectiveness, as suggested by the greater uptake of HIV testing reported by theoretically based interventions included in this review. Although the majority of studies in this review reported a theoretical basis for their intervention, the limited number of theory-based, HIV-focused digital interventions have been noted by others [85,86]; this may be attributed to the speed of development and proliferation of digital approaches to improving HIV outcomes. The findings presented here suggest that theoretical grounding is an important component of effective interventions and should be prioritized in future development.

The findings of this review should be considered with the following limitations. First, due to the restrictions we placed on study design, the interventions included in this review reflect only those conducted as research projects and may not reflect the real-world application of digital technology, including interventions that were not formally evaluated or represented in the published literature. Second, despite our finding that the digital interventions included in this review had a positive overall effect on HIV testing uptake among MSM and TW, the findings do not necessarily reflect the actual quality of the content delivered or levels of end-user acceptability, which are likely to interact in important ways with intervention impact. Third, TW participants were underrepresented in the included studies. TW may use social and sexual networking apps less frequently than MSM, which may reflect the limited number of social and sexual networking sites specifically catering to TW relative to MSM [87]. However, factors such as TW’s reported reliance on online sources of sexual health information [88], perceived acceptability of digital approaches to HIV prevention [43], and examples of real-world applications of digital communication technologies to HIV prevention among TW [87] suggest that TW also stand to benefit from digital approaches to HIV prevention and their inclusion in future trials should be prioritized. Finally, the majority of studies were conducted in high- or middle-income settings. Although the use of digital technology to advance HIV prevention priorities in low-resource settings has been both recommended and applied [64,89], further research is warranted to assess the impact of digital interventions of HIV testing among MSM and TW in these settings.

HIV testing is a key focus of global HIV prevention efforts among MSM and TW, yet multiple barriers continue to prevent levels and frequency of testing required to facilitate the early detection of undiagnosed HIV and initiation of treatment. Digital communication technologies are now an accepted medium for HIV prevention efforts; this review provides further evidence of the role of such technologies in increasing HIV testing uptake among MSM and TW. The inclusion of intervention features such as direct facilitation of HIV testing, involvement of end users in the design process, interactivity, and delivery through the existing mainstream social media platforms may enhance the overall impact and maximize the contribution of digital communication technologies to advancing HIV prevention priorities among MSM and TW.

## Appendix

Multimedia Appendix 1 Ovid Medical Literature Analysis and Retrieval System Online, or MEDLARS Online search strategy.

Multimedia Appendix 2 Study quality appraisal.

## Abbreviations

MeSH: Medical Subject Headings
MSM: men who have sex with men
RCTs: randomized controlled trials
RR: risk ratio
STI: sexually transmitted infection
TW: transgender women
