# Supplementary material for: Using Digital Communication Technology to Increase HIV Testing Among Men Who Have Sex With Men and Transgender Women: Systematic Review and Meta-Analysis
Source: J Med Internet Res. 2020 Jul 28;22(7):e14230. doi: 10.2196/14230 (PMC7420634; doi:10.2196/14230)
Supplement: Multimedia Appendix 1 [file jmir_v22i7e14230_app1.docx]

| 1. male homosexuality/ |
| --- |
| 2. men who have sex with men/ |
| 3. (gay and bisexual men).mp. |
| 4. MSM.mp. |
| 5. 1 or 2 or 3 or 4 |
| 6. Human immunodeficiency virus/di, pc [Diagnosis, Prevention] |
| 7. HIV test/ |
| 8. HIV test*.mp. |
| 9. digital technology.mp. |
| 10. Internet/ |
| 11. internet sex/ |
| 12. online.mp. |
| 13. social media/ |
| 14. ehealth.mp. |
| 15. smartphone/ |
| 16. facebook.mp. |
| 17. geosocial networking app*.mp. |
| 18. online dating/ |
| 19. mobile application/ |
| 20. online dating*.mp. |
| 21. grindr.mp. |
| 22. manhunt.mp. |
| 23. jack'd.mp. |
| 24. growlr.mp. |
| 25. blued.mp. |
| 26. 9 or 10 or 11 or 12 or 13 or 14 or 15 or 16 or 17 or 18 or 19 or 20 or 21 or 22 or 23 or 24 or 25 |
| 27. 6 or 7 or 8 |
| 28. 5 and 26 and 27 |
| 29. limit 28 to (english language and yr="2010 -Current") |

**Appendix 1 Ovid Medline Search strategy**
