# Supplementary material for: Using Digital Communication Technology to Increase HIV Testing Among Men Who Have Sex With Men and Transgender Women: Systematic Review and Meta-Analysis
Source: J Med Internet Res. 2020 Jul 28;22(7):e14230. doi: 10.2196/14230 (PMC7420634; doi:10.2196/14230)
Supplement: Multimedia Appendix 2 [file jmir_v22i7e14230_app2.docx]

**Appendix: Table 6; Study quality appraisal**

| *Study reference* | ***Components rating*** | | | | | | ***Global rating*** |
| --- | --- | --- | --- | --- | --- | --- | --- |
|  | *Selection Bias* | *Study Design* | *Confounders* | *Blinding* | *Data collection method* | *Withdrawals and drop outs* |  |
| Blas 2010 [[47](#_ENREF_47)] | Strong | Moderate | Strong | Weak | Moderate | Moderate | Moderate |
| Blas 2014 [[48](#_ENREF_48)] | Moderate | Strong | Strong | Weak | Moderate | Strong | Moderate |
| Bauermeister 2015 [[46](#_ENREF_46)] | Moderate | Strong | Strong | Weak | Moderate | Strong | Moderate |
| Hirschfield, 2012 [[49](#_ENREF_49)] | Strong | Strong | Strong | Moderate | Weak | Weak | Weak |
| Ko 2013 [[50](#_ENREF_50)] | Strong | Strong | Moderate | Moderate | Moderate | Strong | Strong |
| Patel 2016 [[51](#_ENREF_51)] | Strong | Moderate | Strong | Weak | Moderate | Moderate | Moderate |
| Rhodes 2016 [[53](#_ENREF_53)] | Moderate | Moderate | Weak | Weak | Weak | Weak | Weak |
| Rhodes 2011 [[52](#_ENREF_52)] | Weak | Moderate | Weak | Weak | Moderate | Strong | Weak |
| Tang 2016 [[54](#_ENREF_54)] | Moderate | Moderate | Weak | Weak | Moderate | Weak | Weak |
| Wang 2018[[56](#_ENREF_56)] | Moderate | Moderate | Strong | Weak | Moderate | NA | Moderate |
| Washington 2017 [[55](#_ENREF_55)] | Strong | Moderate | Strong | N/A | Weak | NA | Moderate |
| Young 2015 [[58](#_ENREF_58)] | Strong | Moderate | Strong | Strong | Weak | Strong | Moderate |
| Young 2013 [[57](#_ENREF_57)] | Moderate | Strong | Strong | Weak | Moderate | Strong | Moderate |
